# Release of Major Peanut Allergens from Their Matrix under Various pH and Simulated Saliva Conditions—Ara h2 and Ara h6 Are Readily Bio-Accessible

**DOI:** 10.3390/nu10091281

**Published:** 2018-09-11

**Authors:** Stef J. Koppelman, Mieke Smits, Monic Tomassen, Govardus A.H. de Jong, Joe Baumert, Steve L. Taylor, Renger Witkamp, Robert Jan Veldman, Raymond Pieters, Harry Wichers

**Affiliations:** 1 Food Allergy Research and Resource Program, Department of Food Science & Technology, University of Nebraska, 279 Food Innovation Center, Lincoln, NE 68588-6207, USA; jbaumert2@unl.edu (J.B.); staylor2@unl.edu (S.L.T.); 2 Research Group Innovative Testing in Life Sciences and Chemistry, University of Applied Sciences, Heidelberglaan 7, 3584 CS Utrecht, The Netherlands; mieke.smits@hu.nl (M.S.); robertjan.veldman@hu.nl (R.J.V.); raymond.pieters@hu.nl (R.P.); 3 Food & Biobased Research, Wageningen University and Research, Bornse Weilanden 9, P.O. Box 17, 6700 AA Wageningen, The Netherlands; monic.tomassen@wur.nl (M.T.); harry.wichers@wur.nl (H.W.); 4 TNO, Utrechtseweg 48, 3704 HE Zeist, The Netherlands; aard.dejong@wur.nl; 5 Department of Human Nutrition, Wageningen University and Research, Stippeneng 4, 6708 WE Wageningen, The Netherlands; renger.witkamp@wur.nl; 6 Institute for Risk Assessment Sciences, Faculty of Veterinary Medicines, Utrecht University, Yalelaan 1, 3584 CL, Utrecht, The Netherlands

**Keywords:** peanut, *Arachis hypogaea*, allergen, bio-accessibility, saliva

## Abstract

The oral mucosa is the first immune tissue that encounters allergens upon ingestion of food. We hypothesized that the bio-accessibility of allergens at this stage may be a key determinant for sensitization. Light roasted peanut flour was suspended at various pH in buffers mimicking saliva. Protein concentrations and allergens profiles were determined in the supernatants. Peanut protein solubility was poor in the pH range between 3 and 6, while at a low pH (1.5) and at moderately high pHs (>8), it increased. In the pH range of saliva, between 6.5 and 8.5, the allergens Ara h2 and Ara h6 were readily released, whereas Ara h1 and Ara h3 were poorly released. Increasing the pH from 6.5 to 8.5 slightly increased the release of Ara h1 and Ara h3, but the recovery remained low (approximately 20%) compared to that of Ara h2 and Ara h6 (approximately 100% and 65%, respectively). This remarkable difference in the extraction kinetics suggests that Ara h2 and Ara h6 are the first allergens an individual is exposed to upon ingestion of peanut-containing food. We conclude that the peanut allergens Ara h2 and Ara h6 are quickly bio-accessible in the mouth, potentially explaining their extraordinary allergenicity.

## 1. Introduction

Peanuts (*Arachis hypogaea* L.) are widely consumed in Western countries, Asia, and Africa. The current top three countries in peanut production are China, India, and the USA. The peanut plant thrives in tropical and subtropical climates, preferring well-drained sandy soils, and is cultivated within a wide geographical area [1]. Although peanut is consumed raw or boiled in some cultures, roasting is a preferred processing step because it improves microbial stability, provides a longer shelf life, and also improves the organoleptic properties. Roasted peanuts are commercially available as snack peanuts and as food ingredients or food products such as peanut flour and peanut butter. Peanut consumption in the USA has increased by about 30% since the 1990s, when producers started to highlight the health and nutritional benefits of peanut consumption [1]. Of the different peanut market types, Runner is the dominant type in the USA and is mainly used for the production of peanut butter and peanut flour. The smaller kernel size makes Runner less suitable as snack peanut than, for example, Virginia peanuts, that are larger [2].

While peanuts fit well in a healthy diet, some consumers are allergic to peanuts. The prevalence of peanut allergy is rising in the Western Countries, as recently reviewed [3]. There is no therapy available, and the standard of care is dietary avoidance of peanuts and treatment of allergic reactions by rescue medication [4]. The mechanism of sensitization to peanut is not completely understood, but, clinically, peanut allergy may already develop in infancy, even before the first known consumption of peanuts [5]. Apparently, exposures to traces of peanut can induce sensitization. One source of such traces of peanut is the home environment, where peanut residues are found on floors, table surfaces, and furniture, even after standard household cleaning [6]. Upon ingestion, the peanut is transported through the gastro-intestinal tract for digestion, but, interestingly, some digestion-resistant peanut proteins appear in the blood serum in a relative intact form [7] and may reappear in the saliva even several hours after ingesting peanut [7,8]. For this reason, it has been speculated that a kiss could transfer peanut allergens, representing another form of exposure that may cause sensitization [9].

Recent studies indicate that for children at a high risk of developing food allergies, early oral exposure to peanut may prevent the development of peanut allergy [10,11]. Studies are ongoing to further explore this preventive approach [12] and examine if early oral exposure is also beneficial for other food allergens, although this approach is not always successful [13]. Apparently, oral exposure to allergens is complex in terms of its immunological effects. Because the oral mucosa is the first immune organ that encounters large amounts of food allergens upon ingestion, the bio-accessibility of allergens at this stage may be a key determinant for the development of peanut allergy. In order to be exposed to the oro-esophageal mucosa in the relative short time that food proteins are in the mouth and esophagus, the food proteins should be well bio-accessible. Presently, it is not known how peanut proteins behave in the mouth; therefore, we set out to investigate the release of peanut proteins from a peanut ingredient in the saliva conditions. An improved understanding of the localization of the release of peanut proteins and of the initial exposure of mucosal and epithelial surfaces to peanut proteins may be pivotal to a better comprehension of the development of peanut allergy.

Peanut is a complex biological product that consists of lipids (49%), proteins (25%), carbohydrates (16%), and some moisture and ash [14]; peanut allergens may not be immediately bio-accessible when peanut-containing food is consumed. In many studies on peanut allergens, however, the peanut allergens are used in solubilized form, and this may not be representative of the exposures experienced by human consumers upon ingestion of peanut-containing food. In this study, we aim to investigate the bio-accessibility of peanut allergens under saliva conditions, in order to understand the exposure of the oro-esophageal mucosa to peanut allergens.

## 2. Materials and Methods

### 2.1. Peanut Materials

Light roasted peanut flour, partially defatted to a residual 12% of fat, was obtained from Golden Peanut and Tree Nut Company (Alpharetta, GA, USA). This material has a protein content of 50% *w*/*w*. The purified peanut allergens, namely, Ara h1, Ara h2, Ara h3, and Ara h6, were obtained from lyophilized stock preparations and were used for the identification of bands on sodium dodecyl sulphate-polyacrylamide gel electrophoresis (SDS-PAGE), as described earlier [15,16]. For the calculation of the percentage recovery of individual peanut allergens, the mean *w*/*w* percentages of these allergens in Runner-type peanuts were used [16], taking into account the protein content of the flour (50%) and of intact peanuts [17].

### 2.2. Screening Peanut Protein Solubility in a Wide pH Range (pH 1.5 to 9.0)

An amount of 0.5 (±0.002) g of peanut flour (PF) was suspended in 15 mL of buffer and mixed for 90 min at room temperature. Subsequently, the mixture was centrifuged at 1700 g at room temperature for 5 min, and the supernatant was collected and centrifuged again at 2660 g (for 10 min, then the resulting supernatant was once again mixed for 20 min). The supernatants were stored for analysis. The following buffers were used for extraction: pH = 1.5, 32 mM HCl; pH = 2.6, 21.6 mM Na_2_HPO_4_ + 89.2 mM citric acid; pH = 3.0, 40.8 mM Na_2_HPO_4_ + 79.6 mM citric acid; pH = 4.0, 77.2 mM Na_2_HPO_4_ + 61.4 mM citric acid; pH = 5.0, 102.8 mM Na_2_HPO_4_ + 48.6 mM citric acid; pH = 6.0, 128.5 mM Na_2_HPO_4_ + 35.8 mM citric acid; pH 7.2 to pH 9.0, 50 mM TRIS adjusted with HCl to the target pH. After mixing the peanut flour with medium, the pH was checked and if necessary readjusted to the target value. Protein concentrations were determined using the bicinchoninic acid assay (Sigma, Zwijndrecht, The Netherlands) with bovine serum albumin (BSA) as a standard. The samples were diluted at least 10-fold; the different dissolution buffers had a negligible effect on the assay using this dilution. The protein profiles of the samples were analyzed by SDS-PAGE, essentially as described earlier [16], using 10–20% gradient gels at reducing conditions. The gels were stained with Coomassie Brilliant Blue R250. The samples were analyzed at least two times. Where protein concentrations were above 1 mg/mL, dilutions were made to 1 mg/mL prior to SDS-PAGE analysis.

### 2.3. Testing Peanut Protein Solubility in Saliva Conditions

A saliva-like buffer was made essentially according to Crea et al. [18], containing 8.85 mM NaCl, 7.75 KCl, 11.45 mM NaHCO_3_, 4.18 mM NaH_2_PO_4_, 2.83 mM Na_2_CO_3_, pH = 7.8. The buffers for the pH range 6.5–8.5 were in 50 mM phosphate, using different ratios of monosodium phosphate and disodium phosphate to achieve the target pH, essentially following an online buffer calculator [19].

An amount of 0.5 (±0.002) g of PF was suspended in 10 mL of buffer, pre-warmed to 37 °C. In a first series of experiments, the pH of the mix of peanut flour and buffer was measured and readjusted to the target values with a stock solution of sodium hydroxide. The hydroxide equivalent required was specific for each buffer and appeared highly constant in different experiments. This amount of hydroxide was added to the mixes in further experiments. After mixing the peanut flour with the buffer as described above, samples were taken at 2, 6, and 20 min, and the supernatants were separated from the pellets by centrifugation (30 s at 16,161 g). The supernatants and the pellets were stored for analysis by SDS-PAGE, using 10% gels, in reducing conditions. Fixed volumes of supernatant samples were applied on the gels to allow quantification. The gels were stained with Coomassie Brilliant Blue R250. Quantification was done by densitometry using ImageLab^TM^ software (version 4.1, Bio-Rad, Veenendaal, The Netherlands) with known amounts of marker proteins (BioRad Precision Plus Standard) serving as calibrators. The gel analyses were performed at least twice. For quantitation, the means of two determinations were used. To evaluate the linearity of the quantifications by densitometry, the pH 7.9 extract was tested undiluted (relative concentration = 100%) and diluted to 67%, 44%, 30%, 20%, 13%, 9%, 6%, and 4%. The densitometry signal was linearly dependent on the concentration (*R*^2^ = 0.95). At the lowest concentrations (4%, 6%, 9%), the bands of the least abundant allergens, i.e., Ara h2 and Ara h6, could not be quantified because of their low intensity. The linearity up to the higher concentration ranges indicated that differences in the most intense bands could still be detected and that the densitometry had not yet reached saturation. The staining sensitivity of peanut proteins may be different from that of the marker proteins used as calibrators. While this may impede an absolute quantification (in mg), the used approach is suitable for comparing samples quantified in the same way.

## 3. Results and Discussion

### 3.1. Screening the pH Effect on the Efficiency of Peanut Protein Extraction

**Figure 1:**
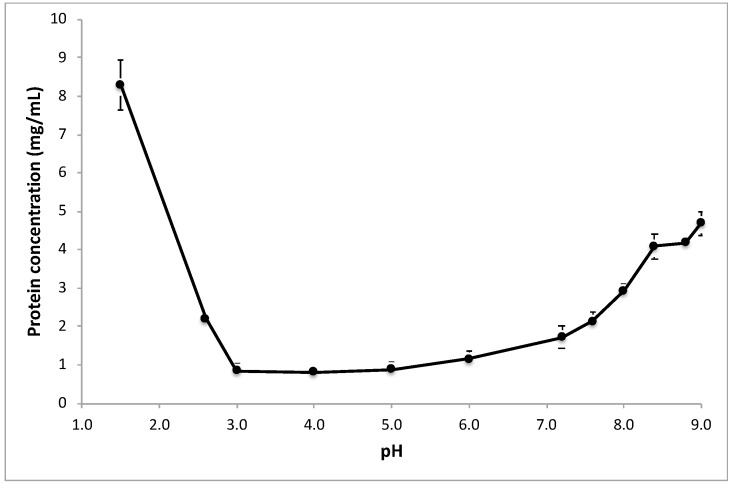
Protein release from light roasted peanut flour at various pHs. Extraction was conducted for 90 min at room temperature. Protein concentrations are shown, as measured with the BCA method (mean values of triplicate experiments; the bars show SD).

Extraction media covering a wide range of pH were used to screen the effect of the extraction pH on the protein content and protein profile of peanut flour extracts. Figure 1 shows a clear minimum efficiency in the extraction of proteins between pH 3 and pH 6. Both at lower pH values and higher pH values, protein extractability became higher, in particular at a pH corresponding to the typical stomach conditions.

Such minimal protein extractability has been shown for several other species of the legume plants [20,21] and has been described in detail for soy [22,23]. The iso-electric point of the abundant proteins in legume seeds is in the range from 4 to 5 [24], and, at this pH, a protein has limited charge and therefore low polarity, which leads to poor solubility in aqueous media. In fact, iso-electric precipitation is industrially used to manufacture soy protein concentrates and isolates [25]. While peanut protein concentrates or isolates are not commercially available, the poor solubility of peanut proteins at pH between 3 and 6 has been shown earlier [26]. Using the extraction ratio (1:30 *w*/*v*) and taking into account the protein content of the peanut flour (50%), the theoretical maximal protein concentration is 16.7 mg/mL. The highest protein concentration we found was about 8.5 mg/mL at pH 1.5. This concentration corresponds to a recovery of about 50%. At a high pH, we found a protein concentration of about 5 mg/mL, which corresponds to about 30% recovery. Thus, even at an extremely low pH and at moderately high pH, protein extraction is not complete in the chosen extraction conditions (90 min at room temperature). It may be that a higher pH would lead to higher extraction recoveries, but pH > 9 is not physiological and was not tested. We included a pH as low as 1.5 because this can reflect the stomach pH. The fact that not all proteins were recovered in the extract is not surprising, as it is known that (light) roasting decreases peanut protein extractability [27].

**Figure 2:**
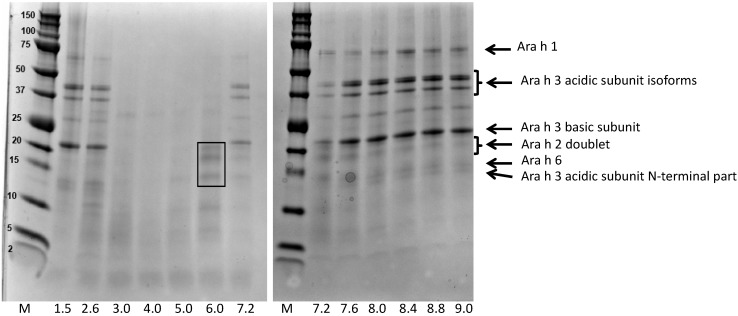
Protein profiles of extracts of light roasted peanut flour prepared at various pHs. Extraction was conducted for 90 min. Protein profiles determined by sodium dodecyl sulphate-polyacrylamide gel electrophoresis (SDS-PAGE), under reducing conditions, on 10–20% gradient gels). The line at the bottom shows the extraction pHs (M refers to the marker proteins, indicated in the left margin in kDa). The arrows at the right point to major allergens. The rectangle in the lane corresponding to pH = 6.0 indicates the Ara h2 doublet (top) and Ara h6 band (bottom).

Apart from the overall protein extractability, we were interested to determine if the pH during the extraction would impact protein composition. Figure 2 shows the protein profiles of the different extracts. In order to facilitate band comparisons, all extracts were diluted to 1 mg/mL, except for the extracts at pH 3, 4, and 5, whose concentrations were already slightly lower than 1 mg/mL. At extremely low pHs and at moderately high pHs, the typical protein band pattern of peanut extracts was observed. In a recent study, we investigated the protein profile of different peanut types and assigned bands using purified individual peanut proteins, in particular the allergens Ara h1, Ara h2, Ara h3, and Ara h6, which together represent about 90–95% of the protein content of a peanut kernel [16]. All these bands were well visible at extremely low pHs and at moderately high pHs. In contrast, at neutral and slightly acidic pHs (3 to 7.6), the bands corresponding to Ara h3 and, to a lesser extent, Ara h1, were far less clear. The bands corresponding to Ara h2 and Ara h6 remained extractable over a wider pH range, although they were poorly visible at pH 4 and 5.

Interestingly, at pH 6 the protein profile of the extract indicated a strong enrichment in Ara h2 and Ara h6. Thus, at this pH, Ara h2 and Ara h6 were highly bio-accessible, while the other allergens Ara h1 and Ara h3 were not. At pH 1.5, a condition similar to that of the human stomach, the protein extractability was high, and all major allergen bands were present. This suggests that in the stomach, all allergens from light roasted peanut flour become bio-accessible.

Centrifugation was used to separate the soluble parts of the extracts (supernatant) from the insoluble parts (pellet). We cannot exclude that some undissolved material or soluble aggregates appeared inadvertently in the supernatants, but, if any, this would be very limited, given the low recoveries of extraction at pH 3 and 4 (Figure 1) and the absence of Ara h1 band in extracts at these pHs (Figure 2).

Poms et al. [28] tested the effect of pH on peanut protein extraction and the reactivity of peanut extracts in commercial enzyme-linked immune-sorbent assays (ELISAs). Interestingly, one ELISA protocol that utilized pH 6.7 as the extraction pH showed poor recovery, while the other ELISA protocols, all using extraction pHs of 7.4 or higher, showed better recoveries. It is not known whether this difference was solely due to the lower extraction pH or whether other factors played a role as well. The specificity of the used ELISA tests was not known at the time of this study, but a recent paper showed that the commercial ELISA kits used by Poms [28] essentially recognize Ara h3 [29], the protein whose extractability appeared low at pHs from 6 to 7.2 in our experiments. A more recent study by Sathe et al. [30] showed that the recovery of proteins by extraction from peanuts was about two times higher at pH 8.45 than at pH 7.2, and that in particular Ara h3 showed a different solubility. Our data are also in line with the work of Walczyk et al. [31], who showed that peanut protein solubility is low at pH 4.75 and increases at pH ≥ 8.5.

### 3.2. Bio-Accessibility of Peanut Allergens in Artificial Saliva

**Figure 3:**
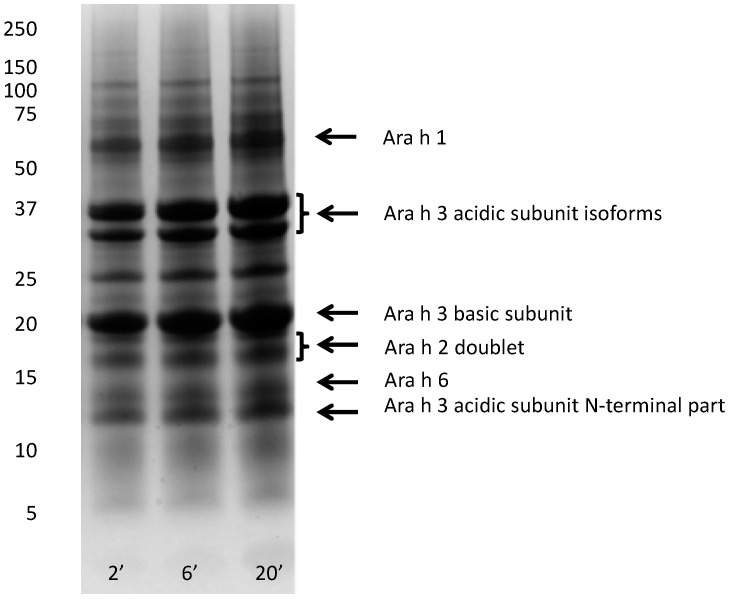
Protein profiles of light roasted peanut flour extracted with artificial saliva (pH = 7.8). Extraction time up to 20 min. Protein profiles were determined by SDS-PAGE, under reducing conditions, on 10% gels. The molecular weight marker is indicated on left in kDa, numbers at the bottom indicate extraction time (minutes). Arrows on the right margin point to major allergens.

**Figure 4:**
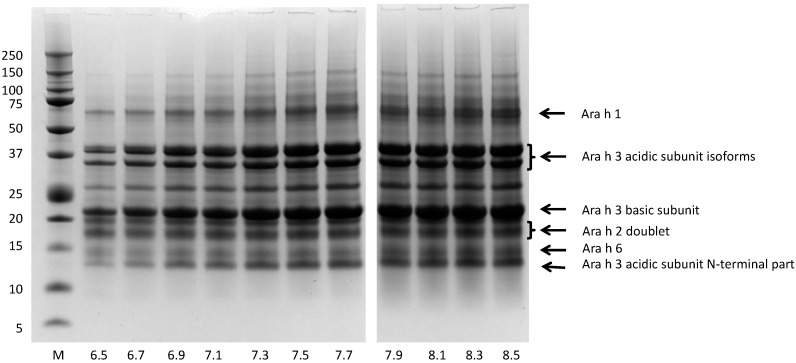
Protein profiles of light roasted peanut flour released at various pHs mimicking saliva. Extraction time 20 min. Protein profiles determined by SDS-PAGE, under reducing conditions, on 10% gels. The molecular weight marker is indicated on the left in kDa. Numbers at the bottom indicate the pH of the extraction buffer. Arrows on the right margin point to major allergens.

The production, composition, and pH of saliva is influenced by many different factors, including mastication. At resting conditions, the dominant saliva-producing glands are the submandibular and sublingual glands, while upon stimulation such as by chewing, the parotid glands take over the majority of saliva production [32,33]. Saliva produced by the parotid glands has a higher carbonate concentration than saliva produced by the submandibular and sublingual glands, which induces an increase in pH upon chewing. In resting conditions, saliva pH ranges normally from 6.5 to 7.5, while the pH may rise to above 8 under chewing conditions [33]. On the basis of the literature and experimental data, Crea et al. [18] proposed a composition for artificial saliva. To investigate peanut protein dissolution under saliva conditions, we used the essential elements of this composition and set the pH at 7.8 to mimic the chewing conditions. Figure 3 shows the protein profiles of artificial saliva extracts of peanut flour prepared with 2, 6, and 20 min of extraction at 37 °C. This time course was short to mimic the exposure in the mouth and during transportation through the esophagus to the stomach. In order to meet the first (2 min) time point, the centrifugation step was kept short, and this could potentially result in the presence of some undissolved material or soluble aggregates in the supernatant samples. The observation that, in some conditions (such as at pH 6.5, see Figure 4), hardly any Ara h1 was visible on SDS-PAGE indicated that this effect was limited. Using dissolution in artificial saliva, protein bands of all allergens (Ara h1, Ara h2, Ara h3, and Ara h6) were clearly visible, even though the extraction time was short. In fact, already at 2 min, the earliest possible time point because of the time needed for sampling and centrifuging, intense bands were observed for these allergens. Increasing the extraction time from 2 to 6 to 20 min only moderately increased the intensity of the protein bands. This suggests that peanut allergens are readily available in the mouth very soon after ingestion of peanut flour, when saliva pH is relatively high.

There is limited research published on the release of peanut allergens in saliva. One study showed that Ara h1 could be detected in saliva 5 min after the ingestion of peanut butter [8]. However, in that study the saliva samples were extracted before quantification of Ara h1, and this extraction step may have released Ara h1 from undissolved peanut material in the sample, leading to an overestimation of the Ara h1 solubility. To our knowledge, no other published data exist on the release of peanut or other allergens in saliva.

### 3.3. The pH of the Extraction Medium Can Be Influenced by Peanut Flour

An important difference between the in vivo situation corresponding to human ingestion of peanut flour-containing food products and that realized through our extraction model is that our model is based on a fixed volume of extraction medium per amount of peanut flour. In vivo, the salivary glands will be stimulated to compensate for the saliva absorbed by the food in the mouth. In our extraction model, the peanut flour may change the pH as a consequence of the buffering effect of the proteins present in peanut flour. To investigate to what extent this occurs, the pH of a range of buffers mimicking saliva (pH range from 6.5 to 8.5) was measured after preparing the buffer and after mixing the peanut flour with the buffer (Table 1). For buffers with pH < 7, a small acidifying effect was observed (less than 0.2 pH units). However, for buffers at higher pHs, the acidifying effect was stronger, up to 0.8 pH units. The amounts of hydroxide needed to re-adjust the pH increased as a consequence of the increasing pH of the extraction medium.

This observation may have implications for protein dissolution studies in general, as the targeted pH is not maintained after mixing protein powders with the extraction medium and may lead to false conclusions when a change in pH is not acknowledged or not adjusted. Our data showed that the acidifying effect was quick and that, as soon as the pH was readjusted, it remained stable (Table 1). For further experiments described in this paper, the required amount of hydroxide was added to the mix of peanut flour and buffer to adjust the pH to the target value.

### 3.4. Bio-Accessibility of Peanut Allergens at the Normal pH Range of Saliva

Because saliva pH can vary depending on mastication activity, we evaluated the effect of pH (range 6.5 to 8.5) on the dissolution of peanut allergens. Figure 4 shows that, at the higher end of the range, intense bands, corresponding to Ara h1, Ara h2, Ara h3, and Ara h6, were recovered in the extracts. A gradual decrease of band intensity was observed when decreasing the pH from pH 7.5 to 6.5 (Figure 4).

**Figure 5:**
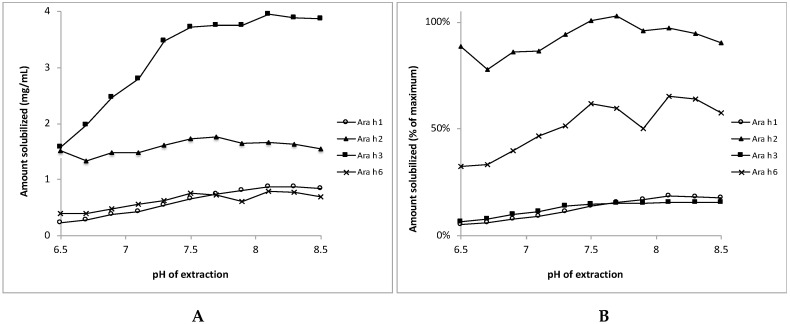
Release of individual peanut allergens from their matrix of light roasted peanut flour dissolved at various pHs mimicking saliva. Panel **A**: Protein concentrations in the supernatants, in mg/mL. Panel **B**: Protein concentrations in the supernatants expressed as percentage of the theoretical maximum value. Mean values of two experiments are shown (SDs were typically between 0 and 8% of the mean (except in one case were the SD was 13% of the values; for Ara h6 at pH = 7.9). SDs are not shown here to improve readability.

The bands of Ara h3 were more prone to this pH-dependent decrease than the bands of the other allergens, which is in line with the difference in extractability of Ara h3 between pH 7.2 and 8.45 reported by Sathe et al. [30]. Using densitometry, the amount of each allergen was determined in extracts prepared at the different pH conditions. Figure 5 shows that, at pH 6.5, the concentrations of Ara h1, Ara h2, Ara h3, and Ara h6 were 0.24, 1.52, 1.58, and 0.40 mg/mL, respectively, and these concentrations increased with increasing extraction pH, in particular for Ara h3, but also for Ara h1 and Ara h6. The recoveries for Ara h1 and Ara h3 were low at pH 6.5, i.e., 5% and 6% of the theoretical maximal values, respectively (Figure 5B). These recovery percentages increased to 18 and 15% for Ara h1 and Ara h3, respectively, when the extraction pH was elevated to 8.5. The recovery of Ara h2 was around 100% at all saliva pHs, and the recovery of Ara h 6 increased from about 35% to 65% with increases in the pH.

Because Ara h2 and Ara h6 are highly similar in biochemical characteristics, it was expected that they would behave the same in the extraction studies; however, the recoveries for Ara h6 were somewhat lower than those for Ara h2. Nevertheless, it is clear that at the lower end of the saliva pH range, the solubility of Ara h2 and Ara h6 was substantially higher than that of Ara h1 and Ara h3. At the higher pHs, the recovery of Ara h1 and Ara h3 increased, yet the recovery percentages were still much lower than those of Ara h2 and Ara h6.

**Figure 6:**
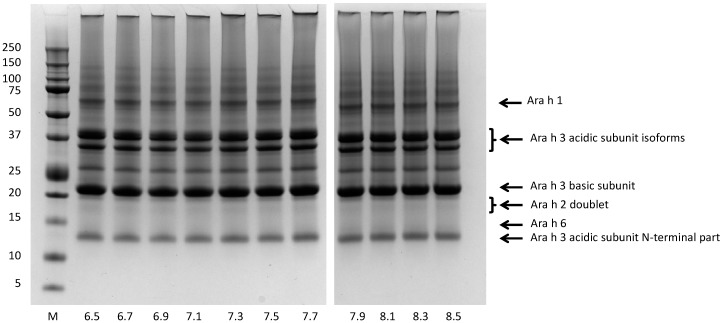
Protein profiles of light roasted peanut flour pellets after extraction at various pHs mimicking saliva. Extraction time 20 min. Protein profiles determined by SDS-PAGE, under reducing conditions, on 10% gels. The marker is indicated on the left in kDa. The numbers at the bottom indicate the pH of the extraction buffer. Arrows on the right margin point to major allergens (note that the area indicted by Ara h2 and Ara h6 is empty indicating no residual Ara h2 and Ara h6 in the pellets.

In addition to investigating the extracted material, we also investigated the protein profile of the non-dissolved material. By boiling the non-dissolved residues remaining after extraction in buffer containing the strong detergent SDS and a reducing agent to break disulfide bonds, we could recover proteins from the residues left over after the dissolution experiments. Figure 6 shows that, for all extraction pHs, the typical bands of Ara h1 and Ara h3 were in the pelleted fraction, together with some aggregated material visible at the top of the lanes.

The presence of intense bands for Ara h1 and Ara h3 in the pellet material is in line with the observation that only a small part of Ara h1 and Ara h3 was extracted (less than 20%; Figure 5B). In the pellet material, no Ara h2 bands were observed, and, for Ara h6, only a vague band was seen at the lowest tested pH conditions (Figure 6). This is in line with the high recovery percentages of Ara h2 and Ara h6 (Figure 5B).

### 3.5. Implications for Peanut Allergy Research

We have investigated the release of peanut allergens from peanut flour in various conditions mimicking human saliva. To date, it is not known how peanut allergens behave in the oro-esophageal area, where the first contact between food proteins and the mucosal immune system takes place. Because peanut is an extraordinary potent allergen, with some of its major allergens being more potent than others, we hypothesized that different peanut allergens my possess different bio-accessibility to the first site of contact, i.e., the mouth, thereby giving rise to different sensitization characteristics.

We selected light roasted peanut flour because it is a commonly used food ingredient and it can represent other heat-treated peanut products in terms of roasting degree. Furthermore, it is available as a well-characterized material and has been used in many peanut allergy studies because of its high lot-to-lot consistency [27,34,35].

Our data showed that Ara h2 and Ara h6 were readily released from the matrix at common saliva pH values (6.5 to 8.5), while Ara h1 and Ara h3 were poorly released. Increasing the pH to higher values reflecting the chewing conditions somewhat increased the release of Ara h1 and Ara h3, but still the recovery levels were low compared to those of Ara h2 and Ara h6. At a lower saliva pH (pH 6), in situations of low production of saliva [33], the extracted proteins from peanut were strongly enriched in Ara h2 and Ara h6. Although this pH does not reflect an active chewing situation, it may represent the situation at the first bite during consumption, i.e., the moment where food is indeed exposed to such pH. The fact that the peanut flour has an acidifying effect on the dissolution media suggests that this low pH situation in the mouth may be maintained for a period of time. Thus, Ara h2 and Ara h6 may be the first peanut allergens that individuals are exposed to upon ingesting peanut or peanut-containing foods.

Collectively, our data suggest that Ara h2 and Ara h6 are highly bio-accessible in the mouth and esophagus and may trigger immunological effects in the oro-esophageal mucosa. The difference in solubility between Ara h2 and Ara h6 on the one hand, and Ara h1 and Ara h3 on the other, is not due to different isoelectric points, because all four allergens have isoelectric points between 5 and 6. The solubility difference may be determined by their molecular weight and quaternary structure. Ara h2 and Ara h6 are small (15–20 kDa) monomeric proteins [36,37], while Ara h1 and Ara h3 are larger and form complexes up to 180–700 kDa and 360–380 kDa, respectively [38,39,40]. Upon roasting, Ara h1 and Ara h3 may aggregate, further limiting their solubility, while this is not the case for Ara h2 and Ara h6 [41]. For Ara h 1, denaturing media such as high-molarity urea are needed for optimal solubilization [31]. Indeed, when we suspended the undissolved material in a strongly denaturing buffer (SDS-containing buffer with a reducing agent) for SDS-PAGE analysis, we recovered Ara h1 and Ara h3 bands as well (Figure 6). These remarkable differences in size and quaternary organization of peanut allergens may partially explain why Ara h2 and Ara h6 are more soluble than Ara h1 and Ara h3.

A limitation of our study is that we have not investigated if the solubility of peanut proteins in the mouth and esophagus indeed leads to enhanced sensitization potency. Investigating this would require in vivo experiments, for example using a mouse model and applying oral sensitization [42], but this was beyond the scope of the current work.

Ara h2 and Ara h6 are the most potent peanut allergens [43,44], and the presence of IgE to these two allergens is the best predictor of clinical peanut allergy [45,46]. The vast majority of peanut-allergic patients have IgE to Ara h2 or Ara h6 or both [34]. In part, the potency of Ara h2 and Ara h6 may be explained by their resistance to digestion in the human gastrointestinal tract. It has been shown that Ara h2 and Ara h6 are more resistant against digestion with pepsin and trypsin than Ara h1 and Ara h3 [47,48] and survive the gastrointestinal conditions relatively unaffected [49]. Our data showing that Ara h2 and Ara h6 are highly bio-accessible in the mouth (and esophagus) suggest that these allergens have the unique opportunity to interact with the mucosal immune system before reaching the stomach, which may lead to different sensitizing potential in comparison to other peanut allergens. This may provide an additional explanation for the extraordinary allergenicity of Ara h2 and Ara h6 compared to other peanut allergens.

## 4. Conclusions

We show that allergens Ara h2 and Ara h6 are quickly released from peanut matrix at saliva conditions. This makes them bio-accessible in the mouth and esophagus, and enable them to interact with the oro-esophageal mucosal immune system, potentially explaining their extraordinary allergenicity compared to other peanut allergens.

## Figures and Tables

**Table 1:** Influence of peanut flour dissolution on the pH of the extraction buffer.

Theoretical pH	Measured pH of Buffer	pH after Peanut Flour Addition *	OH^−^ Equivalent Added (mM)	pH Immediately after Adjusting	pH 2 min after Adjusting
6.5	6.49	6.42	2.5	6.53	6.50
6.7	6.69	6.57	3.0	6.69	6.69
6.9	6.88	6.76	3.0	6.88	6.87
7.1	7.08	6.95	4.0	7.09	7.08
7.3	7.28	7.10	4.8	7.28	7.28
7.5	7.48	7.23	4.8	7.52	7.53
7.7	7.67	7.33	5.5	7.70	7.69
7.9	7.87	7.48	5.5	7.90	7.87
8.1	8.09	7.55	6.0	8.11	8.10
8.3	8.29	7.65	6.3	8.30	8.29
8.5	8.49	7.69	6.5	8.49	8.48

* Peanut flour addition: 500 ± 2 mg.
